# Inkjet Printing of PEDOT:PSS Based Conductive Patterns for 3D Forming Applications

**DOI:** 10.3390/polym12122915

**Published:** 2020-12-04

**Authors:** Indranil Basak, Gudrun Nowicki, Bart Ruttens, Derese Desta, Jeroen Prooth, Manoj Jose, Steven Nagels, Hans-Gerd Boyen, Jan D’Haen, Mieke Buntinx, Wim Deferme

**Affiliations:** 1 Hasselt University, Institute for Materials Research (IMO-IMOMEC), B-3590 Diepenbeek, Belgium; indranil.basak@uhasselt.be (I.B.); bart.ruttens@uhasselt.be (B.R.); derese.desta@uhasselt.be (D.D.); jeroen.prooth@uhasselt.be (J.P.); manoj.jose@uhasselt.be (M.J.); steven.nagels@uhasselt.be (S.N.); hansgerd.boyen@uhasselt.be (H.-G.B.); jan.dhaen@uhasselt.be (J.D.); 2 IMEC vzw-Division IMOMEC, Wetenschapspark 1, B-3590 Diepenbeek, Belgium; 3 Institute for Materials Research, Packaging Technology Center, IMO-IMOMEC, Hasselt University, Wetenschapspark 27, 3590 Diepenbeek, Belgium; gudrun.nowicki@uhasselt.be (G.N.); mieke.buntinx@uhasselt.be (M.B.)

**Keywords:** inkjet printing, stretchable electronics, 3D forming of touch sensor

## Abstract

This paper presents the formulation, inkjet printing, and vacuum forming of a conductive and stretchable polymer, poly(3,4-ethylenedioxythiophene) polystyrene sulfonate (PEDOT:PSS), ink on a stretchable and transparent thermoplastic polyurethane (TPU) substrate. The formulation of the conductive and stretchable ink is achieved by combining PEDOT:PSS with additional solvents, to achieve the right inkjet properties for drop-on-demand (DoD) inkjet printing. A conductive pattern can be printed from the 21 µm orifice on a flexible and stretchable TPU substrate, with a linewidth down to 44 µm. The properties of the printed pattern, in terms of sheet resistance, morphology, transparency, impact of weather conditions, and stretching are investigated and show sheet resistances up to 45 Ohm/sq and transparencies as high as 95%, which is comparable to indium tin oxide (ITO). Moreover, in contrast to ITO, one-time stretching up to 40% can be achieved, increasing the sheet resistance up to 214 Ohm/sq only, showing the great potential of this ink for one-time stretching. Finally, as a proof of this one-time stretching, the printed samples are vacuum formed around a 3D object, still showing sufficient conductivity to be applied as a capacitive touch sensor.

## 1. Introduction

The most attractive transparent conductive electrode applied in many organic electronic applications is indium tin oxide (ITO). This layer is mostly used in optoelectronics devices (e.g., organic light emitting diodes, solar cells, etc.), because it is highly conductive and transparent up to 90%. It is, however, (a) expensive, (b) a hard but fragile material, and (c) it requires high temperatures to produce. For these reasons, ITO cannot be used for stretchable electronic applications [1]. Different types of materials such as graphene [2,3], carbon nanotubes [4,5], conductive polymers [6,7,8,9], and metal nanowires [10,11,12] have been investigated to replace ITO. Mainly, these materials suffer from a high electrical resistance and poor elasticity. These challenges restrict their application in stretchable applications such as wearable electronics and 3D forming. Highly conductive, transparent, and stretchable materials are essential for stretchable electronics to be integrated in different devices with new form factors, such as textile, skin-based, and wearable devices [13,14].

Stretchable conductive traces have been fabricated in recent research in different ways, strain engineering on the one hand and the formulation of nanocomposites or the use of semi-conductive polymers on the other hand [15,16,17,18]. In the first approach, a conductive structure, often from non-stretchable materials, is deposited on a pre-strained elastomer substrate. A buckled structure is achieved when the stretch is released. In this pre-strain-release-buckling strategy, the critical part is to pre-strain the substrate, presenting a challenge towards roll-to-roll manufacturing [19,20]. Another approach is to print a horse-shoe pattern on a stretchable substrate, which will behave like a spring when stretched [21]. In the second approach, rigid filler materials with good electrical properties are dispersed into elastomeric materials. As a conductive filler material, nanomaterials with a high aspect ratio (nanowires and nanotubes) have been shown to be the best candidate. In these cases, the resulting composites were strain sensitive, even up to more than 100%. Hu et al. stretched silver nanowires (Ag NW) on cross linked poly(acrylate) by 50%; the sheet resistance was increased 2.3 times [22]. Another study showed an increase in sheet resistance of Ag NW on polydimethylsiloxane (PDMS) by a factor of 1.9 when stretched by 100% [23]. Recently, Ag NW was inkjet printed with a 50 µm nozzle on an octadecyltrichlorosilane (OTS) pre-treated glass substrate. Afterwards, it was transferred to PDMS. The achieved minimum average resistance was 63.73 Ω [24]. Ag NW nanocomposites are promising materials, which show similar behaviour to ITO, in terms of conductivity and transparency [25,26,27,28,29,30].

The conducting polymer (poly(3,4-ethylenedioxythiophene) polystyrene sulfonate—PEDOT:PSS) is attractive because it is a readily solution-processable formulation that could be patterned using digital printing technology. Further, by tuning the molecular structure, electrical and mechanical properties of the polymer-based formulation can be altered. Large-scale production of flexible electronics is possible, due to the solution processability. However, high conductivity and stretchability have not been achieved simultaneously [31,32,33]. Different types of mass printing techniques have been developed to print a pattern with high resolution, high stability, low cost, and low waste of materials. The printing techniques, mainly inkjet [34], screen [35], and gravure printing [36], have been gaining more and more attention for fabricating organic and structural electronics. The physical properties of the inks, especially viscosity, surface tension, and concentration of the conductive fillers, are different for each technology. Screen and gravure printing are mostly compatible with high viscous inks and thus perfectly suitable for Ag NW-based formulations. Inkjet printing requires inks with low viscosity and high surface tension and fillers with a size below 500 nm [37]. Inkjet printing gets special attention in printed electronics because of its simplicity of fabrication, easily changeable digital print patterns, and because it is a non-contact and maskless patterning technique. In addition, it is time and cost efficient. Printing technology, however, encounters a transformational change in the manufacturing of flexible and stretchable electronics. Therefore, it is necessary to fabricate high performance stretchable inks by selecting the printing parameters meticulously to achieve formulations that can be printed with inkjet printing on stretchable substrates and that maintain their properties upon stretching.

Stable formulations from PEDOT:PSS for inkjet printing have been reported in the literature. However, it should be mentioned that these inks were formulated for printing on flat and rigid or flexible substrates and not for stretching applications. Polymeric light emitting diodes (PLED) were fabricated on a rigid glass substrate and PEDOT:PSS was printed as an anode layer [38]. López et al. developed an PEDOT:PSS ink that was suitable for inkjet printing with a DoD Dimatix 2800 inkjet printer from Fujifilm and the layer achieved a resistivity of 0.15 Ω.cm, corresponding to a conductivity of 6.6 S/cm [38]. Recently, multi-electrode arrays were inkjet printed on a flexible polyimide substrate with an in-house PEDOT:PSS ink. The impedance of each PEDOT:PSS electrode was measured to be 19.5 kΩ at 1 KHz. In addition, these electrodes were stable for three weeks [39]. Recently, PEDOT:PSS was inkjet printed for photovoltaic applications [40]. However, its application towards a stretchable and transparent conductor for 3D forming has not been discussed in literature.

In this work we develop a stretchable conductive polymer-based inkjet ink for printing with a DoD Dimatix 2800 inkjet printer (Santa Clara, CA, USA). Special attention is given to the fact that the final printed pattern should be stretchable up to at least 40%, to be able to use it for 3D forming. The PEDOT:PSS ink was inkjet printed on a stretchable and transparent thermoplastic polyurethane (TPU) substrate and the final printed pattern was characterized for its final properties of sheet resistance, transparency, morphology, impact of weathering, and stretching. The stability and jettability of the ink was optimized to prevent clogging of the printer nozzle. Finally, 3D forming using vacuum forming technology was achieved for the first time as a proof of the one-time stretching behaviour of the printed pattern, and the printed PEDOT:PSS structure was applied for a touch sensing application.

## 2. Experimental

**Figure 1:**
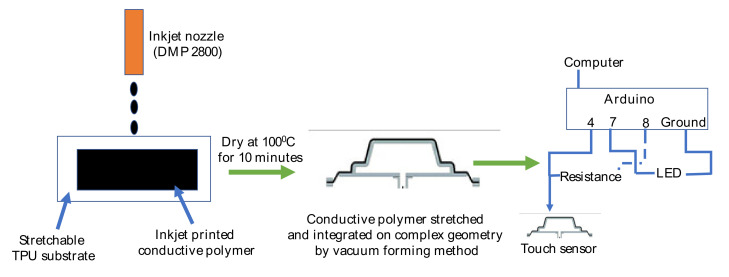
Schematic diagram of inkjet printed touch sensor.

In this study PEDOT:PSS named as Clevios™ PH1000 from HERAEUS (Hanau, Germany) was applied as the conductive polymer. The solid content in water is 1–1.3%, the boiling point approximately 100 °C, the pH value 1.5–2.5, and the viscosity 15–50 mPa·s. The viscosity of the formulation was adjusted using dimethyl sulfoxide (DMSO, Amresco) to print with a Dimatix DMP 2800 inkjet printer. then, 10, 20, and 30 volume% of Clevios™ PH1000 was inkjet printed successfully on thermoplastic polyurethane (TPU). Afterwards, it was stretched and integrated on complex geometry using a vacuum forming method. Stretched and integrated Clevios™ PH1000 shows enough potential for touch sensing applications (Figure 1).

### 2.1. Printing

Drop-on-demand inkjet printing was used to print the conductive patterns. A square (3 × 3 cm^2^) was selected as a test pattern and printed on a transparent and stretchable TPU foil from the company Grafytip (Houthalen, Belgium). The printability was characterized for the ink samples 7, 8, and 9 (see Table 1) with volume percentages of 30%, 20%, and 10% of PEDOT:PSS loading in DMSO, respectively. It was not possible to print with more than 30% PEDOT:PSS due to agglomeration and nozzle clogging (Table 1). Characterization of the rheological properties of the ink are measured using a AR-G2 viscosity measurement setup from TA Instruments (Antwerp, Belgium) for the viscosity, a OCA 15plus contact angle measurement set-up from Dataphysics (Nazareth, Belgium ) for the surface tension, and a volumetric measuring device from Hirschmann (Eberstadt, Germany ) for the density. The volume percentages of 10%, 20%, and 30% PEDOT:PSS loaded ink could be printed without blocking the orifice after storing at 5 °C for more than three months, two months, and two weeks, respectively. The printing stage temperature was set at 40 °C and the drop spacing was set to 15 µm. Rectangles, having 1 up to 5 consequent layers on top of each other, were printed to decrease the sheet resistance. After printing, the test patterns were dried on the printing stage for 15 min and then annealed in an oven at 100 °C for 10 min. In the case of stacked layers, all layers were annealed together within an oven after printing. To test the linewidth and morphology of the printed patterns, the drop spacing was changed from 10 µm up to 80 µm.

### 2.2. Characterization

Linewidth of the printed pattern was measured using scanning electron microscopy (SEM, Zeiss, Zaventem, Belgium). Sheet resistance of the test pattern was measured using the Van der Pauw, four-probe method, applying the following equation:

(1) $$R_{\mathrm{square}}=\mathrm{cf}\;\times \;\frac{V}{I}$$

where $R_{\mathrm{square}}$ is the sheet resistance, cf is the error correction (cf= 4.53), V is the voltage between the inner probes, and I is the applied current [41,42,43,44]. The morphology and transparency of the printed test patterns were measured using an atomic force microscope (AFM, Park NX10, Park systems, Suwon, Korea) and UV-VIS spectrometer (Cary 5000 UV-Vis-NIR, Agilent technologies, Santa Clara, CA, USA), respectively. Additionally, the impact of weather conditions and stretching behaviour of the patterned sample were measured via exposure in a Q-Sun Xenon climate chamber (Q-SUN Xe-1, Q-LAB, Bolton, UK) and an in-house developed stretch test device, respectively. Vacuum forming was performed with a vacuum forming system (Formech type 450 dt, Leimersheim, Germany) on a cone that was 3D printed with black polylactic acid (PLA) to show the applicability of the formulated ink for 3D forming applications such as touch sensing.

## 3. Results and Discussion

### 3.1. Ink Formulation

Typical physical properties of functional inks, such as rheology, surface tension, and density, can be modified by optimizing the solvent mixture as well as the loading of the functional material into the solvent mixture. 

To print a reliable pattern, it is important to generate stable drops without long tails and satellites. The behaviour of the ink during jetting and droplet flight depends on dimensionless numbers, such as the Reynolds number (Re), Weber number (We), and Ohnesorge number (Oh) [45,46,47]. 

**Figure 2:**
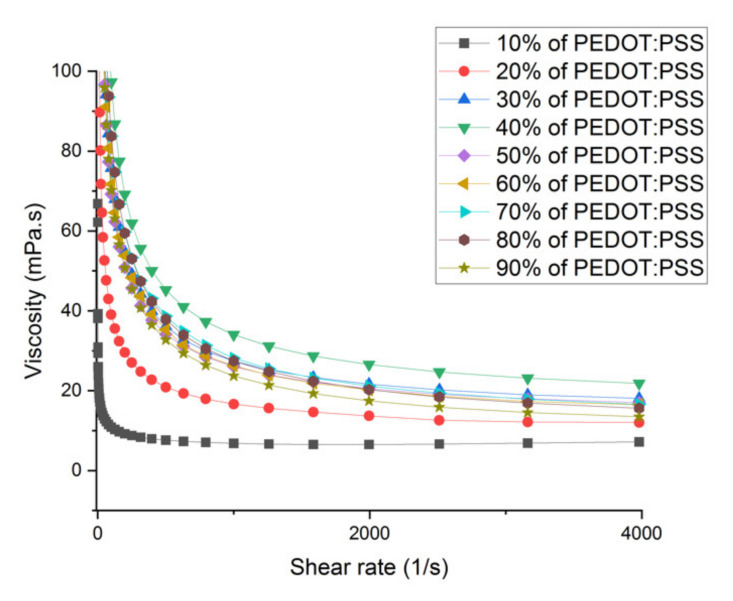
Influence of shear rate on the viscosity of the developed PEDOT:PSS ink showing a constant viscosity value above a certain shear rate.

Usually, the printability is defined by the Z number, which is 1/Oh. A stable drop can be generated when the Z parameter value is in between 1 and 10 [48]. For low values of Z, viscous dissipation does not allow drops to eject, whereas for high values of Z, drops are ejected from the nozzle with a large number of satellite drops. Another challenge for ink formulation is to avoid solute clogging and blockage of the nozzle during printing. The main reasons behind nozzle blocking are oversized solute and evaporation of the solvent in an orifice. The nozzle blocking can be avoided, if the size of the solute is less than 1/10th of the print head orifice diameter [49]. The size of the solute can be reduced by adapting membrane filtering and sonication-driven scission processes [50,51]. Additionally, high boiling point solvents that have low volatility can avoid solvent evaporation in an orifice [52]. In general, the shear rate of inkjet nozzles is in the range of 106 s-1, which is higher than attainable in conventional rheometers [53]. The shear rate of our device was limited up to 4 × 103 s−1. The influence of the shear rate on the viscosity was measured as is shown in Figure 2. It was observed that for 10%, 20%, and 30% of PEDOT:PSS loading, the viscosity was decreased to 7, 12, and 14 mPa.s at shear rates up to 4 × 10³ s−1. From the curve it can be predicted that for higher shear rates, the viscosity will stay rather constant, which means that for the high shear rates applied during DoD inkjet printing, the viscosity measured at 4 ×10^3^ s−1 can be taken as the final viscosity. Table 1 shows the ink composition and corresponding viscosity, surface tension, density, Ohnesorge number, and Z number.

The basic requirements for viscosity, surface tension, and density are 10–11 mPa.s, 30–32 mN/m, and 1 gm/cm^3^, respectively, for our Dimatix 2800 DoD inkjet printer from Fujifilm [54]. Based upon these properties, inks with 10%, 20%, and 30% of PEDOT:PSS loading (Table 1) were selected, and after an ultrasonic bath of 100 min, the inks were filtered using a 0.45 µm PVDF syringe filter, to achieve a size of the solute of less than 1/10th of the printhead orifice diameter, i.e., 21 µm. It was found that the selected inks were inkjet printable without blocking the orifice.

### 3.2. Printability

A uniform and high-resolution pattern can improve the device stability and performance. A high quality pattern is a prerequisite especially for stretchable conductive devices. Optimized flexibility, elasticity, and durability ensure high performance of the device. A high-resolution rectangle pattern was inkjet printed on the stretchable TPU substrates. TPU is a thermoplastic block copolymer that has an excellent elasticity. It can be produced using different methods, e.g., melt processing, extrusion, and injection moulding. Additionally, it has a high wear resistance and good weather stability [55]. The final shape of the printed rectangle depends on droplet-substrate interaction and the drying dynamics of the pattern. 

Droplets coalescence depends on the print head diameter, velocity, pitch, and contact angle of the droplets. If the drop spacing is too large, then drops do not connect with each other. A further decrease in drop spacing, results in drops connecting with each other, but the edges are not smooth and a scalloped line is formed. If drop spacing decreases further, a uniform line with smooth edges is achieved, but with an even further decrease of the drop spacing, a series of periodic bulges, connected by a ridge, are achieved. The width of the ridge is similar to the diameter of an individual printed droplet [56]. In this work, the drop spacing was optimized at 40 µm for the printed lines, as discussed below.

Additionally, the impact of droplets on the substrate depends on the surface free energy of the substrate. The surface free energy of solids cannot be measured directly. It can be measured approximately by measuring the contact angle of a set of liquids that are in contact with the solid. The most popular and straightforward method to measure the surface free energy is Fowkes theory [57]. 

To optimize the wetting of the printed droplets on the substrate of choice, a pre-treatment of the substrate could be performed. This will increase the surface free energy of the substrate such that it better matches the surface tension of the ink. It can be observed from Table 2 that the surface free energy of the TPU was increased after a corona treatment.

### 3.3. Printing Resolution

**Figure 3:**
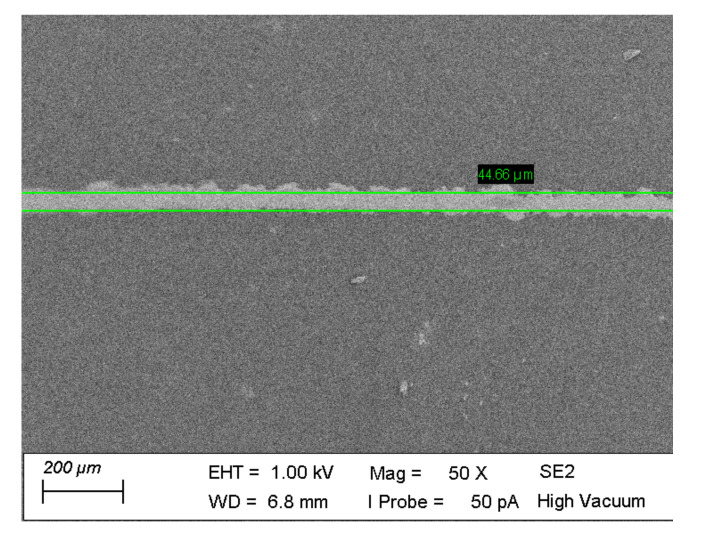
Inkjet printed linewidth with 10% PEDOT:PSS loaded ink.

When one wants to successfully print a high-resolution pattern, it is important to understand the effect of the dot-to-dot spacing. The influence of dot-to-dot spacing on the final printing linewidth has been reported before [58]. To determine the printing resolution, various dot-to-dot spacings were printed with the 10% PEDOT:PSS loaded ink, on a corona treated TPU substrate. Both linewidth and pattern uniformity were strongly influenced by the dot-to-dot spacing. It was found that a uniform line was formed when the dot-to-dot spacing was around 40 µm. The corresponding linewidth was 44 µm (Figure 3). It can be seen that the line still shows some unevenness at the edges, but a further decrease of the drop spacing does not result in smoother edges. The uneven pre-treatment of the substrate before printing can play a detrimental role in this and needs further investigation for optimized edges of the printed patterns.

### 3.4. Opto-Electronic and Morphological Characterization

**Figure 4:**
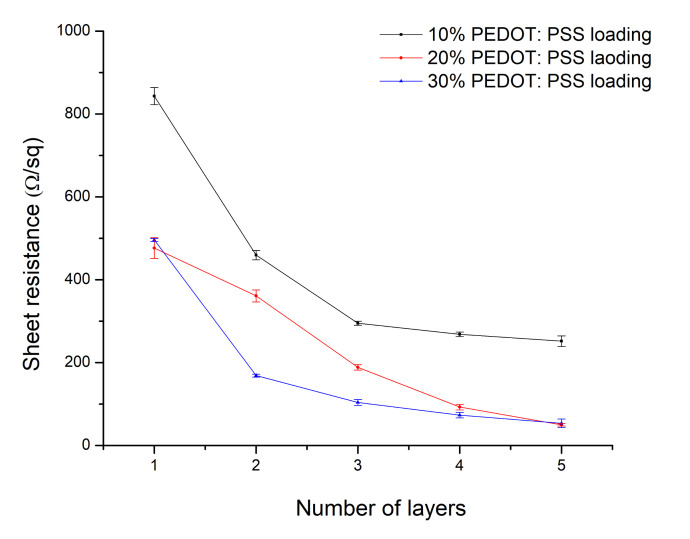
Sheet resistance measurements of inkjet printed layers.

The sheet resistance was measured for samples printed from the 10% and 30% loaded PEDOT:PSS ink. It was found that a sheet resistance of 860 Ω/sq was measured for the 10% PEDOT:PSS loaded ink while the sheet resistance dropped to 490 Ω/sq for the 30% PEDOT:PSS loaded ink. To optimize the conductivity of the printed patterns even further, multiple printing passes (2–5 passes) were performed. The sheet resistance dropped further to 250 Ω/sq for the 10% PEDOT:PSS loaded ink and to 45 Ω/sq for the 30% PEDOT:PSS loaded ink, by printing five layers on top of each other, as is also shown in Figure 3. It was observed that the sheet resistance decreased rapidly from 1 to 3 stacked layers but did not change much after three printed layers (Figure 4).

**Figure 5:**
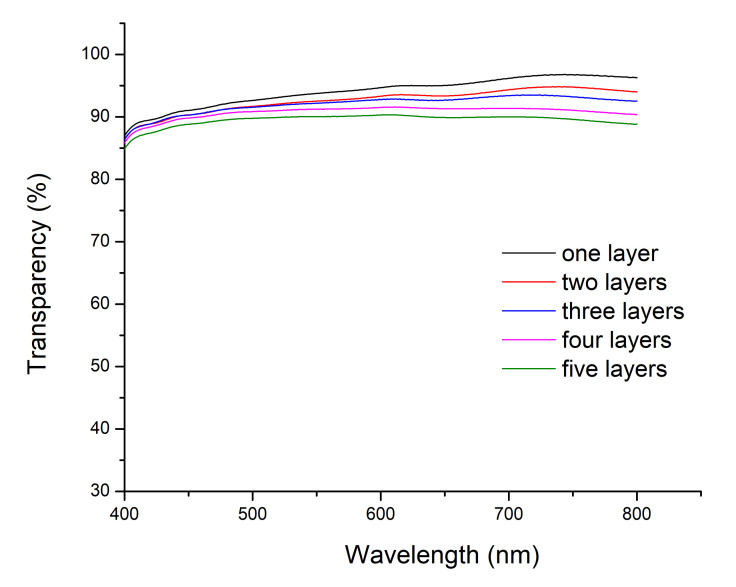
Transparency of inkjet printed layers made using a 10% PEDOT: PSS loading.

UV-VIS spectroscopy was used to measure the transparency of the printed samples. First, the transparency of the TPU blank sample and inkjet printed samples on the TPU substrate were measured. Afterwards, the final transparency of the printed samples were plotted in Figure 5, after subtracting the results of the TPU blank sample. It is shown that the transparency (400–800 nm wavelength region) was slightly decreased by increasing the number of printed layers, as expected. Layers printed from 10% PEDOT:PSS loaded ink show very high transparency (>86%) in the 400–800 nm wavelength region, which is better than that of ITO films. It is also clear from Figure 5 that the PEDOT:PSS layers are more transparent in the higher wavelength regime (600–800 nm), whereas a lower transparency is measured at lower wavelengths, below the absorption edge of PEDOT:PSS. The transmittance of layers printed from 20% and 30% loaded inks show the same trend as that shown in Figure 5, however having of course a lower transparency as again more PEDOT:PSS is deposited (as shown in the Supplementary Information S1).

**Figure 6:**
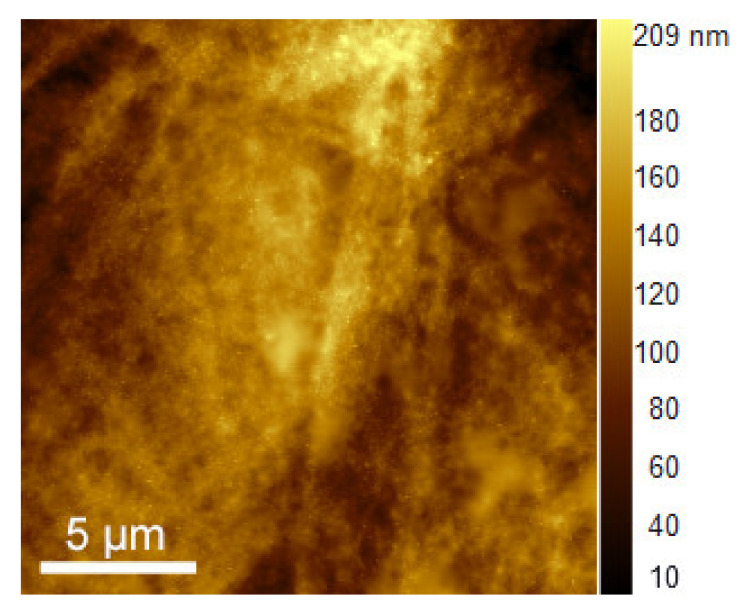
Atomic force microscope (AFM) image of printed structure after 5 passes with the 10% PEDOT:PSS ink.

Finally, the surface roughness of the printed structures was determined from the AFM images shown in Figure 6. It was noticed that the root mean squared surface roughness values remain below 35 nm for all printed samples, going from 10% and over 20% to 30% of PEDOT:PSS loading and with an increasing number of layers, from 1 to 5 (Supplementary Information S2). As a conclusion, we can say that our printed layers are smooth, highly transparent, and show sheet resistance values comparable to other reported PEDOT:PSS-based transparent electrodes.

### 3.5. Influence of UV Exposure on the Opto-Electronic Properties

**Figure 7:**
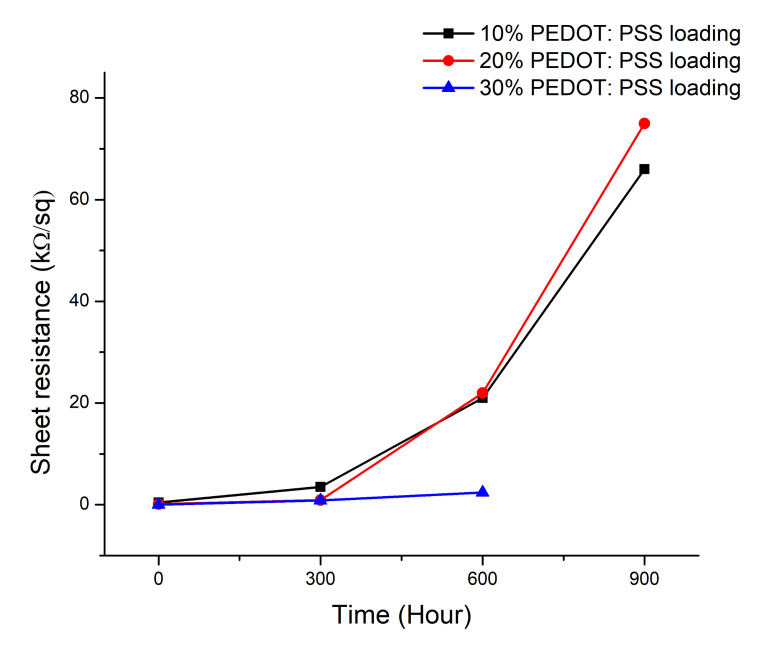
Sheet resistance analysis after Q-Sun Xenon exposure.

Polymers such as PEDOT:PSS start to degrade upon exposure to sunlight [59]. Therefore, it is important to understand the lifespan of the printed ink under natural sunlight. We used 900 light hours at 0.55 W/m^2^ 340 nm (daylight filter), which corresponds to approx. 1 year outside exposure in central Europe. This also corresponds to 2050 MJ exposure (300–800 nm). We exposed the printed samples for 900 h (1782 W/m^2^) in a Q-Sun Xenon test chamber that can produce the full spectrum of sunlight, including ultraviolet, visible, and infrared radiation. Throughout the experiment, the sheet resistance was measured every 300 h. The sheet resistance of inks with 10%, 20%, and 30% of PEDOT:PSS loading was measured for five layers on top of each other. Layers consisting of a high amount of polymer will degrade more as compared to layers with a low amount of polymer. It was found that after 900 h of Q-Sun Xenon exposure, the sheet resistance increased sharply (0.45 KΩ/sq to 66 KΩ/sq) for the ink with 10% of PEDOT:PSS loading. Next, the ink with 20% of PEDOT:PSS loading showed a similar deterioration (0.15 KΩ/sq to 75 KΩ/sq). However, the ink with 30% of PEDOT:PSS loading exhibited the most dramatic deterioration. It showed conductivity only up to 600 h of exposure when sheet resistance increased from 0.04 kΩ/sq to 2.4 kΩ/sq (Figure 7). Therefore, as 30% of PEDOT:PSS loading has more PEDOT:PSS content than the 20% and 10% ink, after 600 h, due to rapid deterioration, sheet resistance measurements were not possible anymore. Photographic images of the printed layer before and after the Q-Sun Xenon exposure showed that the transparency of the printed layers decreased because of deterioration (S3–S5).

### 3.6. Influence of Stretching for 3D Forming Applications

**Figure 8:**
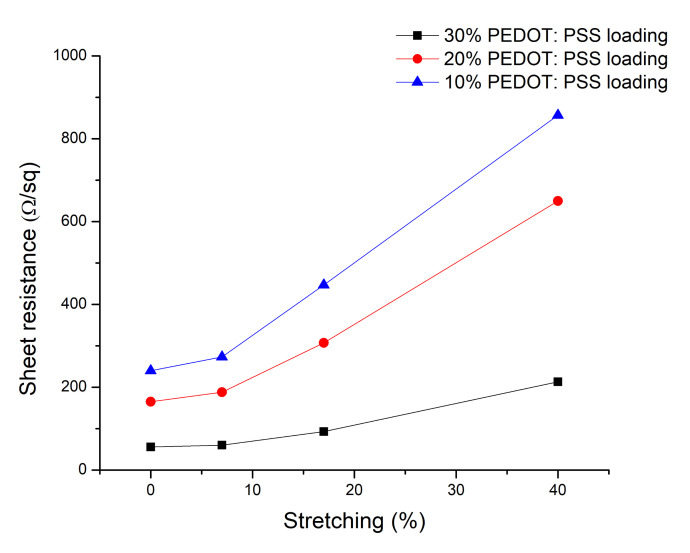
Sheet resistance analysis after one-time stretch cycle.

When the printed layers are applied for 3D forming applications, it is important that the conductivity after stretch is still in the order of kOhms to be able to apply these 3D integrated conductive layers as touch sensors. As the focus is on 3D forming, only a one-time stretch is needed, and no breakage of the printed pattern should occur. We measured the sheet resistance of our printed sample while it was stretched. In this step, we stretched the sample up to 7%, 17%, and 40% respectively with a home-made stretch-test bench. After a one-time stretching of 40%, the sheet resistance was increased from 240 Ω/sq to 857 Ω/sq for the 10% PEDOT:PSS ink, 165 Ω/sq to 650 Ω/sq for the 20% PEDOT:PSS ink, and 56 Ω/sq to only 213 Ω/sq for the 30% PEDOT:PSS (Figure 8).

**Figure 9:**
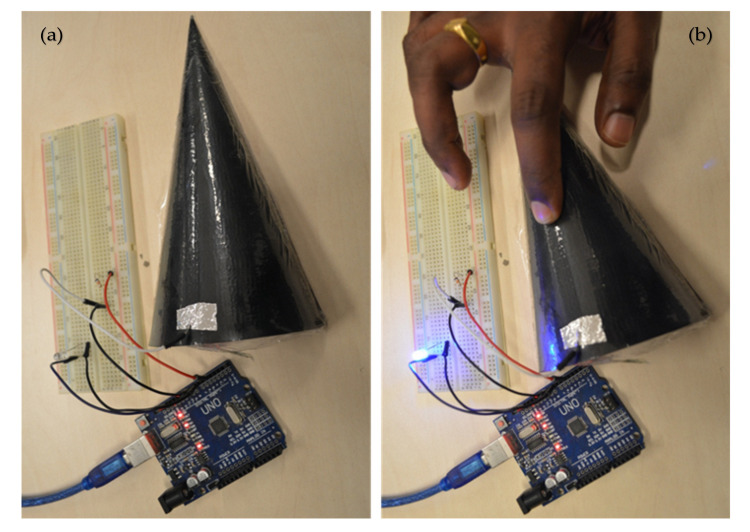
Photo images of vacuum-formed touch sensor, (**a**) without touch, (**b**) LED glows with touch.

For the application of 3D forming, a one-time stretch is enough as these foils will be stretched while forming around a 3D object and will stay in a stretched form after finalisation of the forming process. In general, flexible foils applied for vacuum forming cannot tolerate large strain deformation. A core challenge is to achieve high performance electronic systems that offer lightweight, elastic, and low modulus responses to large strain deformation. Therefore, we inkjet printed a conductive line on a corona treated lightweight, flexible, and stretchable TPU foil that offers large strain deformation (Figure 8). To attach the TPU foil on the 3D cone, some glue was pasted around the edges of the cone. Afterwards, the TPU foil was stretched and integrated on a 3D-printed cone via vacuum forming at room temperature. A touch sensing circuit was built on a bread board and programmed with Arduino. The integrated light emitting diode (LED) on the circuit represents the performance of the touch sensor. The performance of the vacuum-formed touch sensor is captured in Figure 9. 

## 4. Conclusions

In this study, we developed a polymer PEDOT:PSS-based ink formulation that was inkjet printable through a 21 µm orifice. Ink loaded with 10%, 20%, and 30% of PEDOT:PSS was printable without blocking the nozzle. We achieved a minimum linewidth of 44 µm for a drop spacing of 40 µm. We investigated the sheet resistance, morphology, transparency, impact of sunlight, and stretchability of the printed conductive layer. The sheet resistance by printing 5 layers on top of each other reached 45 Ω/sq with a 30% PEDOT:PSS ink. As compared to the literature, similar results were found for PEDOT:PSS and Ag NW. The printed pattern with 10%, 20%, and 30% PEDOT:PSS loading was transparent, although the transparency decreased by printing a number of layers on top of each other. Nonetheless, a very high transparency of 98% was achieved for the 10% PEDOT:PSS ink after printing only 1 layer. As compared to ITO, reaching 45 Ω/sq with a transparency above 90%, this inkjet printed PEDOT:PSS ink shows great promise. Furthermore, it can not only be applied as electrode (replacing ITO) but can also be printed in lines having linewidths down to 44 µm. In terms of stability of the printed layers, it was also found that the sheet resistance was increased (dramatically) after 900 h of Q-Sun Xenon exposure. However, the pattern with 10% and 20% of PEDOT:PSS loading showed conductivity even after 900 h of Q-Sun Xenon exposure in the range of several tens of kΩ/sq. Further, the printed pattern can be stretched up to 40% without breaking and thus reaches its application goal to be integrated with vacuum forming on 3D objects. In terms of transparency and printability, the 10% PEDOT:PSS loaded ink showed the most promising result. On the other hand, in terms of sheet resistance, impact of weather condition, and stretchability, the 30% PEDOT:PSS loaded ink showed very positive results. Additionally, it was noted that the achieved sheet resistance after 40% of strain was still high enough for touch sensing applications. Finally, it can be concluded that these stable polymer-based inks with 10%, 20%, and 30% of PEDOT:PSS loading have the potential to be the formulation of choice when inkjet printing conductive patterns for 3D forming applications such as touch sensing.

## Figures and Tables

**Table 1:** Ink formulation for inkjet printing (Dimatix DMP-2800), showing the rheological parameters of different inks, containing different poly(3,4-ethylenedioxythiophene) polystyrene sulfonate (PEDOT:PSS) vs. dimethyl sulfoxide (DMSO) concentrations.

Sample Number	PEDOT:PSS (vol.%)	DMSO (Vol.%)	Viscosity @22 °C in mPa.s (η)	Surface Tension @ 22 °C in mN/m (γ)	Density in mg/cm^3^ (ρ)	Ohnesorge Number (Oh)	Z Number
1	90	10	15.48	62	1.03	0.42	2.38
2	80	20	15.61	60	1.04	0.43	2.33
3	70	30	16.60	57	1.05	0.47	2.13
4	60	40	16.54	56	1.08	0.46	2.18
5	50	50	17.06	53	1.09	0.48	2.08
6	40	60	21.48	46.5	1.11	0.65	1.54
7	30	70	14.04	49	1.11	0.41	2.44
8	20	80	12.13	46	1.12	0.37	2.70
9	10	90	7.12	44	1.14	0.22	4.55

**Table 2:** Effects of surface treatment on stretchable thermoplastic polyurethane (TPU) substrates.

Surface Treatment	Contact Angle of Water on TPU	Contact Angle of Diiodomethane on TPU	$\sigma_{S}^{D}$ $\left(\frac{mN}{m}\right)$	$\sigma_{S}^{P}$ $\left(\frac{mN}{m}\right)$	$\sigma_{S}=\sigma_{S}^{D}+\sigma_{S}^{P}$ $\left(\frac{mN}{m}\right)$
No treatment	67.5	42.4	38.3	7.3	45.7
Corona treatment	52.1	32.2	43.2	13.4	56.6

## Supplementary Materials

The following are available online at https://www.mdpi.com/2073-4360/12/12/2915/s1, Figure S1: Decrease of transparency analysis of inkjet printed layers with (**a**) 20% and (**b**) 30% PEDOT: PSS loading; Figure S2: AFM images of the inkjet printed PEDOT:PSS pattern after (**a**) 1 pass, (**b**) 2 passes, (**c**) 3 passes, (**d**) 4 passes; Figure S3: 10% of PEDOT:PSS loading: (**a**) one layer and (**b**) five layers. Five layers of 10% of PEDOT:PSS loading after Xenon exposure: (**c**) 300 h, (**d**) 600 h, and (e) 900 h; Figure S4: 20% of PEDOT: PSS loading: (**a**) one layer and (**b**) five layers. Five layers of 20% of PEDOT:PSS loading after Xenon exposure: (**c**) 300 h, (**d**) 600 h, and (e) 900 h; Figure S5: 30% of PEDOT: PSS loading: (**a**) one layer and (**b**) five layers. Five layers of 30% of PEDOT:PSS loading after Xenon exposure: (**c**) 300 h, (**d**) 600 h, and (e) 900 h.
