# Supplementary material for: Inkjet Printing of PEDOT:PSS Based Conductive Patterns for 3D Forming Applications
Source: Polymers (Basel). 2020 Dec 4;12(12):2915. doi: 10.3390/polym12122915 (PMC7762030; doi:10.3390/polym12122915)
Supplement: Supplementary file 1 [file polymers-12-02915-s001.pdf]

polymers

MDPI

Article

Supplementary Materials

Inkjet Printing of PEDOT:PSS Based Conductive Patterns for 3D Forming Applications

Indranil Basak [1], Gudrun Nowicki [2], Bart Ruttens [1], Derese Desta [1], Jeroen Prooth [1], Manoj Jose [1], Steven Nagels [1], Hans-Gerd Boyen [1], Jan D'Haen [1], Mieke Buntinx [2] and Wim Deferme [1,*]

[1] Hasselt University, Institute for Materials Research (IMO-IMOMEC), B-3590 Diepenbeek, Belgium; indranil.basak@uhasselt.be (I.B.); bart.ruttens@uhasselt.be (B.R.); derese.desta@uhasselt.be (D.D.); jeroen.prooth@uhasselt.be (J.P.); manoj.jose@uhasselt.be (M.J.); steven.nagels@uhasselt.be (S.N.); hansgerd.boyen@uhasselt.be (H.-G.B.); jan.dhaen@uhasselt.be (J.D.)

[2] Hasselt University, Institute for Materials Research (IMO-IMOMEC), Packaging Technology Center, IMO-IMOMEC, Hasselt University, Wetenschapspark 27, 3590 Diepenbeek, Belgium; gudrun.nowicki@uhasselt.be (G.N.); mieke.buntinx@uhasselt.be (M.B.)

* Correspondence: wim.deferme@uhasselt.be

Received: 9 November 2020; Accepted: 3 December 2020; Published: 4 December 2020

S1: Transparency Analysis

The transparency of the inkjet printed PEDOT:PSS layer was measured using UV-VIS spectroscopy. The transparency of the inkjet printed PEDOT:PSS on TPU was compared with the blank TPU sample. The final transparency value is shown in Figure S1. It was observed that the transparency value was inversely proportional with PEDOT:PSS loading and directly proportional with the number of printed layers. For the 20% and 30% of PEDOT:PSS loaded ink, the achieved transparency was 95% for one printed layer, but for five printed layers, it was decreased up to 80%. It is clear from Figure S1 that the value of transparency with the number of printed layers was almost constant for both 20% and 30% of PEDOT:PSS loaded inks. One printed layer with 10%, 20%, and 30% of PEDOT:PSS loaded ink shows similar transparency, but for five printed layers, the transparency of 20% and 30% of PEDOT:PSS loaded ink is approximately 10% more as compared to the transparency value of 10% PEDOT:PSS loaded ink.

(a)

(b)

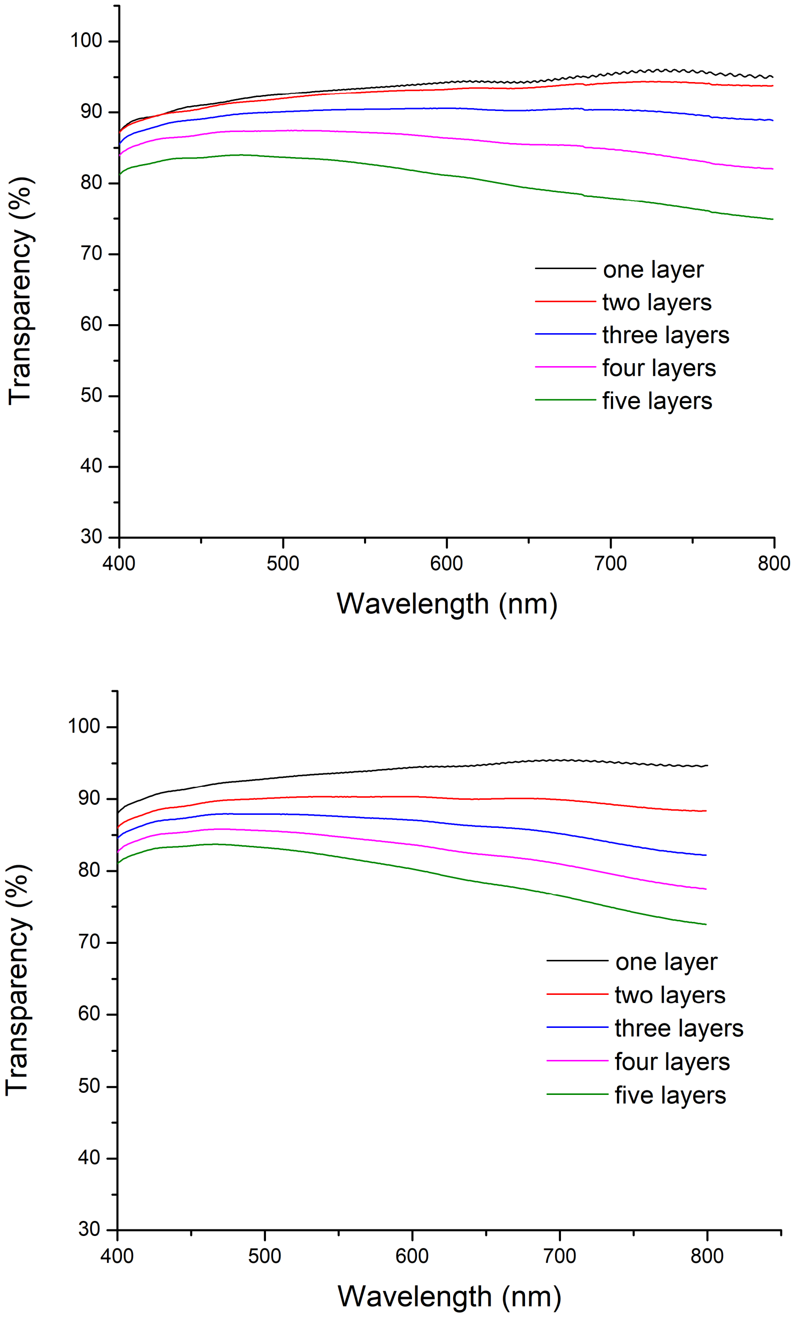

Figure S1. Decrease of transparency analysis of inkjet printed layers with (**a**) 20% and (**b**) 30% PEDOT: PSS loading.

S2: Surface Roughness of Inkjet Printed PEDOT:PSS Pattern

The surface roughness or morphology of the inkjet printed pattern deposited with 10% PEDOT:PSS loading was measured using an atomic force microscope. The pattern was deposited via 1–4 passes. It was observed that for 1, 2, 3, and 4 printing passes the root mean squared surface roughness values were 19.9 nm, 6.8 nm, 6.9 nm, and 12.6 nm, respectively. It was clear that for 1 to 4 passes surface roughness values were below 20 nm. Since the roughness values are below 20 nm, it can be concluded that our inkjet printable PEDOT:PSS based ink can print a smooth and homogeneous layer.

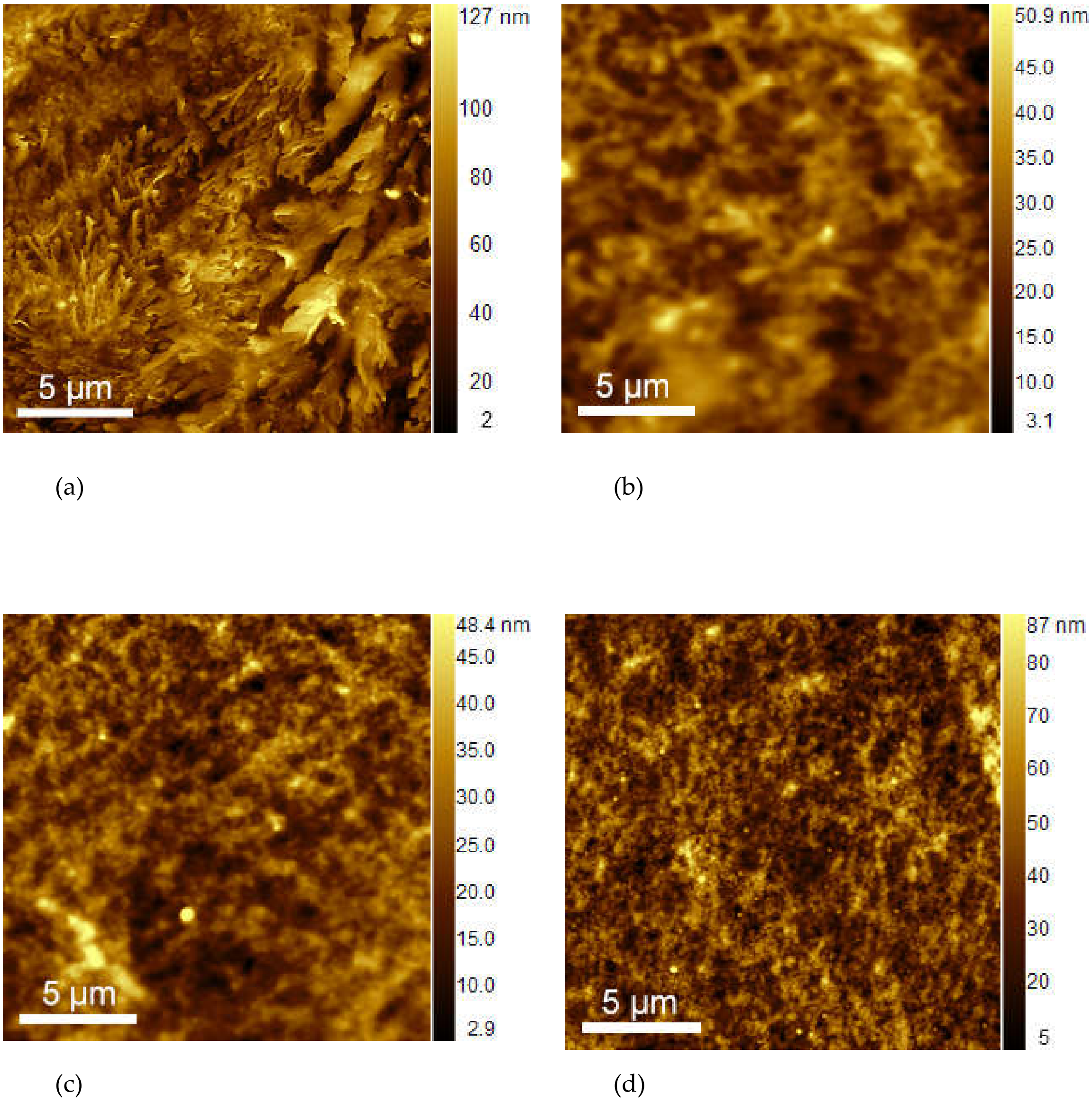

Figure S2. AFM images of the inkjet printed PEDOT:PSS pattern after (**a**) 1 pass, (**b**) 2 passes, (**c**) 3 passes, (**d**) 4 passes.

S3-S5: Inkjet Printed PEDOT:PSS Layer Before and After Q-Sun Xenon Exposure

A 3 × 3 cm2 square was inkjet printed on a TPU substrate after corona surface treatment. Photographic images of printed patterns with 10%, 20%, and 30% of PEDOT:PSS loaded ink are shown in Figure S3a, Figure S3b, Figure S4a, Figure S4b, Figure S5a, and Figure S5b, respectively. It was observed that the darkness of the printed layer was increased with PEDOT:PSS loading and the number of printed layers. For one printed layer it was almost invisible, but the darkness was increased with five printed layers. Furthermore, to optimize the stability, five printed layers of 10%, 20%, and 30% of PEDOT:PSS loaded ink were exposed to natural sunlight that was created artificially using a Q-Sun Xenon chamber. It was clear from the photographic images S3 (c–e), S4 (c–e), and S5 (c–e) that the darkness of the printed layer was increased with the Q-Sun Xenon exposure time (300 h, 600 h and 900 h). Due to Q-Sun Xenon exposure, deterioration of the PEDOT:PSS was increased, which damaged and increased the darkness of the printed samples.

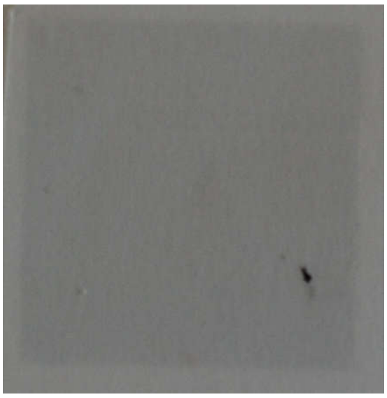

(a)

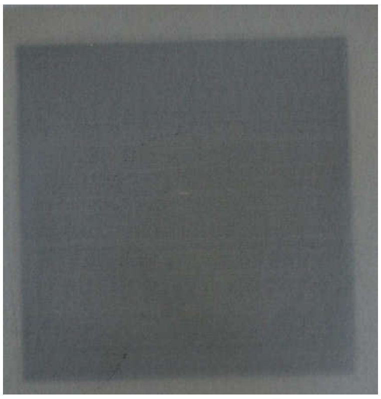

(b)

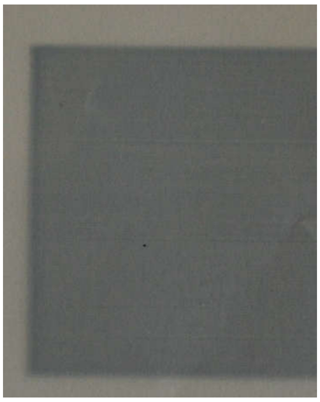

(c)

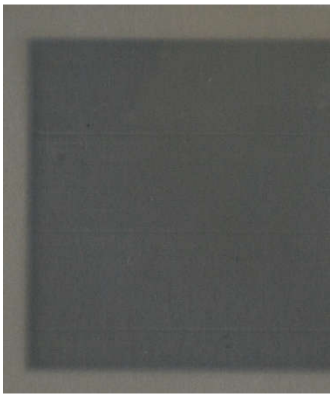

(d)

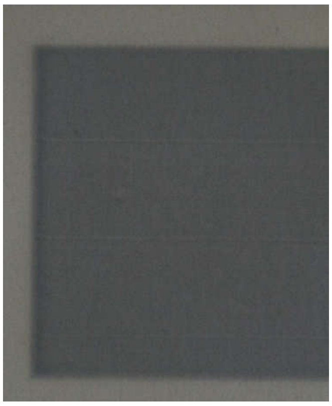

(e)

Figure S3. 10% of PEDOT:PSS loading: (**a**) one layer and (**b**) five layers. Five layers of 10% of PEDOT:PSS loading after Xenon exposure: (**c**) 300 h, (**d**) 600 h, and (**e**) 900 h.

(a)

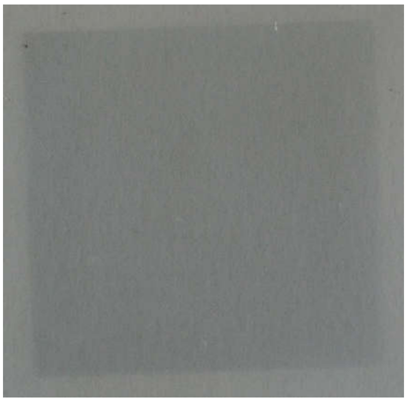

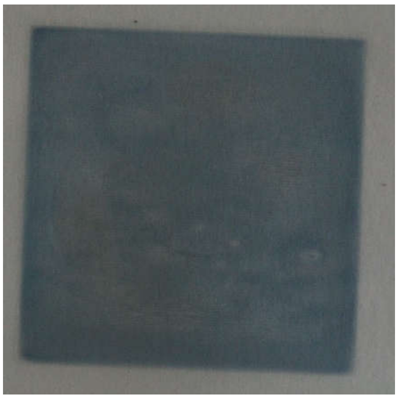

(e)

(c) (d)

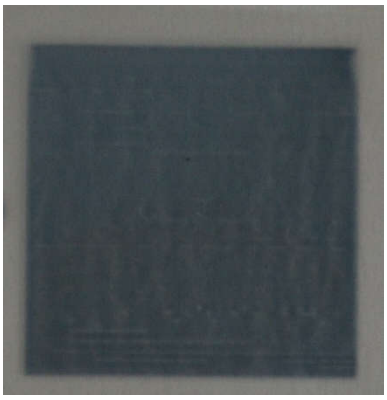
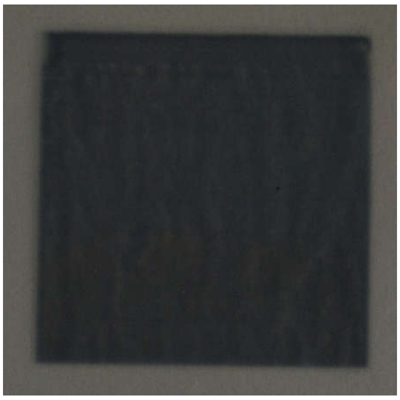
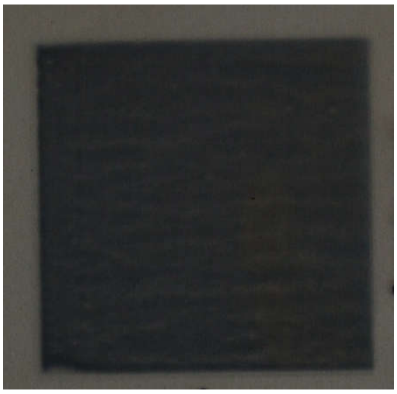

Figure S4. 20% of PEDOT: PSS loading: (**a**) one layer and (**b**) five layers. Five layers of 20% of PEDOT:PSS loading after Xenon exposure: (**c**) 300 h, (**d**) 600 h, and (**e**) 900 h.

(a)

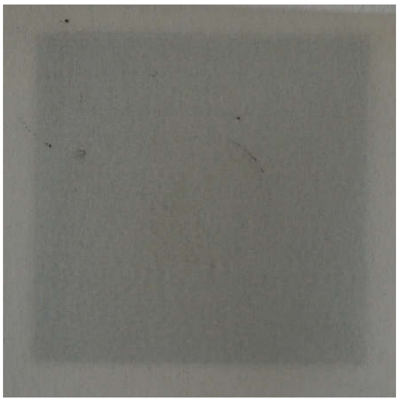

(b)

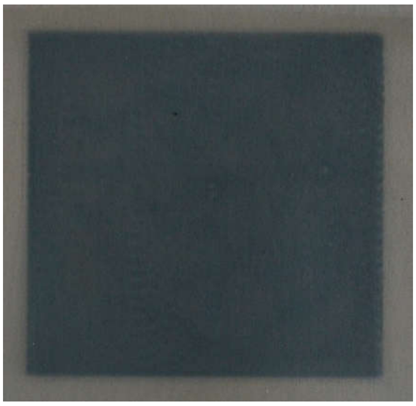

(e)

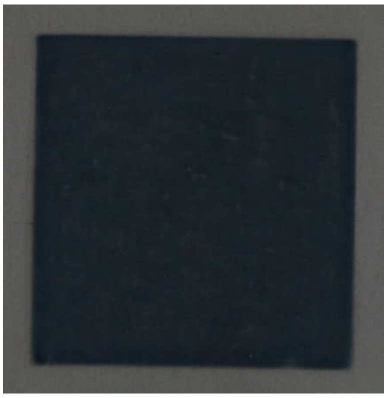
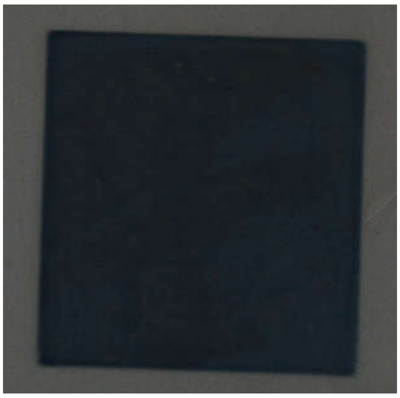
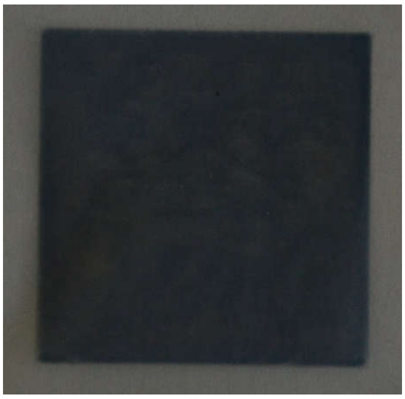

Figure S5. 30% of PEDOT: PSS loading: (**a**) one layer and (**b**) five layers. Five layers of 30% of PEDOT:PSS loading after Xenon exposure: (**c**) 300 h, (**d**) 600 h, and (**e**) 900 h.
